# Living-donor liver transplantation in children with inherited metabolic and genetic cholestatic liver diseases: a single-center retrospective cohort study

**DOI:** 10.1186/s13023-026-04369-4

**Published:** 2026-04-30

**Authors:** Hui Liu, Zhida Chen, Wanfu Li, Halimulati Huxitaer, Gulimiremu Maimaiti, Ayiguzaili Maimaijiang, Yeliaman Jiayilawu, Aerxin Habuding, Runqi Xi, Haoyu Wang, FangJuan Song

**Affiliations:** The First Affiliated Hospital of Xinjiang Medical University, Urumqi Xinjiang , China

**Keywords:** Living donor liver transplantation, Pediatrics, Inherited metabolic liver diseases, Genetic cholestatic liver diseases, Growth and development

## Abstract

**Background:**

Living donor liver transplantation (LDLT) has become an important therapeutic option for children with selected inherited metabolic and genetic cholestatic liver diseases (IM-GCLDs).However, evidence on disease-specific outcomes across different diagnostic categories remains limited, and we therefore conducted a single-center retrospective study with contemporaneous non-IM-GCLD pediatric LDLT recipients as a comparator to better contextualize transplant-related outcomes and disease-specific benefits.

**Results:**

Among 21 children with IM-GCLDs, the median follow-up was 21 months; two patients died (one perioperatively from disseminated intravascular coagulation and one at 21 months from pneumonia-related multiorgan failure), and all others are alive with functioning grafts. Disease-specific manifestations, including neuropsychiatric symptoms, portal hypertension, metabolic crises, cholestasis, hyperbilirubinemia, and hyperammonemia, improved or resolved in almost all survivors.At 6 months after LDLT, in children <10 years, mean weight- and height-for-age Z-scores increased from −0.48 to 0.43 and from −0.76 to −0.01; in children ≥10 years, mean height Z-scores increased from −1.49 to −0.53 while BMI Z-scores showed no significant change. Overall survival did not differ significantly between IM-GCLDs and non-IM-GCLD indications.

**Conclusions:**

Living donor liver transplantation in children with IM-GCLDs not only improves survival but also confers disease-specific benefits, including recovery of neurologic function, metabolic stabilization, relief of portal hypertension and cholestasis, and catch-up growth. These findings support LDLT as an important therapeutic option for IM-GCLDs, while diagnosis-tailored perioperative assessment and long-term management remain essential given the phenotypic heterogeneity.

**Supplementary Information:**

The online version contains supplementary material available at 10.1186/s13023-026-04369-4.

## Introduction

Inherited metabolic and genetic cholestatic liver diseases (IM-GCLDs) are a heterogeneous group of disorders caused by monogenic defects leading to hepatic metabolic derangements and cholestasis [1]. Their clinical manifestations are complex, and a proportion of affected children progress to end-stage liver disease. With advances in pediatric liver transplantation, liver transplantation has become an important therapeutic option for selected IM-GCLDs. IM-GCLDs now constitute the second most common indication for pediatric liver transplantation after biliary atresia [2].

Previous studies have demonstrated that LDLT can markedly improve survival and quality of life in children with IM-GCLDs [3, 4]. However, most reports have focused on single entities such as Wilson disease, hereditary tyrosinemia, or maple syrup urine disease, and their conclusions are therefore limited to specific subtypes. In routine clinical practice, IM-GCLDs encompass diverse hepatic and extrahepatic phenotypes (e.g., neurologic involvement, portal hypertension, metabolic crises, cholestasis, and growth impairment), which may translate into different post-transplant trajectories and long-term quality of life. yet there is a paucity of studies that systematically integrate and compare outcomes across multiple IM-GCLD subtypes within a single pediatric LDLT cohort.

Against this clinical background, we conducted an integrated analysis of children with various IM-GCLD subtypes undergoing LDLT and compared them with contemporaneous non-IM-GCLD recipients.This comparison is clinically important because the non-IM-GCLD cohort underwent the same transplant procedures and perioperative care in the same center and period, serving as an internal benchmark for transplant-related outcomes (survival and complications), while IM-GCLDs are expected to show additional disease-specific benefits (neurologic, metabolic, portal-hypertension, cholestasis, and growth-related improvements). By performing this systematic evaluation, we aim to provide more comprehensive evidence to inform individualized transplant decision-making and long-term management in children with IM-GCLDs.

## Materials and methods

### Study design and patients

We retrospectively reviewed the clinical data of all pediatric patients who underwent living donor liver transplantation (LDLT) in the Department of Pediatric General Surgery at the First Affiliated Hospital of Xinjiang Medical University between January 2021 and May 2025. A total of 45 children received LDLT during this period, including 21 (45.7%) with inherited metabolic and genetic cholestatic liver diseases (IM-GCLDs) and 24 transplanted for non-IM-GCLD indications.In children classified as IM-GCLDs, the diagnosis was confirmed by genetic testing and/or liver biopsy. Only patients with complete perioperative and follow-up data were included in the analysis, and those with incomplete records or loss to follow-up were excluded. The study protocol was approved by the institutional ethics committee (This study was approved by the Ethics Committee of the First Affiliated Hospital of Xinjiang Medical University (approval No. K202602-46). The requirement for informed consent was waived due to the retrospective nature of the study) and written informed consent was obtained from the parents or legal guardians of all participants.

### Donor and transplantation procedures

All donors were biological parents who underwent comprehensive anatomical and genetic evaluation. Graft types included 13 left hemi-liver, 7 left lateral segment, and 1 right posterior segment, with detailed clinical characteristics summarized in Table 1. In cases where the donor was a heterozygous carrier of the causative mutation, donation was deemed safe and acceptable after multidisciplinary assessment.

**Table 1:** Baseline characteristics of pediatric patients

NO.	Age	Sex	Weight (kg)	Diagnosis	Donor/Recipient ABO Type	Graft Type	Complications	Donor Source	Status
1	15ys	M	40	WD	AB(+)/ A(+)	Left lobe	None	Father	Alive
2	11ys	F	37	WD	B(+)/ B(+)	Left lobe	None	Father	Alive
3	1y2mo	M	6.5	HT-Ⅰ	B(+)/ O(+)	Left lateral segment	Pulmonary infection	Mother	Deceased
4	10ys	F	33	CHF	AB(+)/ A(+)	Left lateral segment	Lymphatic leakage	Mother	Alive
5	8ys	M	29	CHF	A(+)/ A(+)	Left lateral segment	None	Father	Alive
6	8ys	M	38	WD	O(+)/ B(+)	Right posterior segment	Chronic rejection	Father	Alive
7	9ys	F	30	WD	B(+)/ B(+)	Left lobe	Disseminated intravascular coagulation	Mother	Deceased
8	6ys	F	23	CHF	B(+)/ B(+)	Left lobe	None	Father	Alive
9	9mo	M	6	MSUD	B(+)/ B(+)	Left lateral segment	None	Father	Alive
10	12ys	F	38	CHF	O(+)/ O(+)	Left lobe	Small-for-size syndrome	Mother	Alive
11	10ys	M	27	CD	A(-)/ A(+)	Left lobe	None	Mother	Alive
12	11ys	M	34	WD	A(+)/ A(+)	Left lobe	None	Mother	Alive
13	9ys	F	30	WD	B(-)/ B(+)	Left lobe	None	Mother	Alive
14	10ys	F	37	WD	AB(+)/ AB(+)	Left lobe	Chronic rejection	Mother	Alive
15	10ys	F	24	GSD-Ⅰ	B(+)/ A(+)	Left lobe	None	Mother	Alive
16	2ys5mo	M	10	HT-Ⅰ	O(+)/ O(+)	Left lateral segment	None	Mother	Alive
17	9ys	M	23	ALGS	O(+)/ O(+)	Left lobe	Pulmonary infection	Mother	Alive
18	12ys	F	39	WD	B(+)/ B(+)	Left lobe	None	Mother	Alive
19	13ys	F	35	NPD-B	O(+)/ O(+)	Left lobe	None	Mother	Alive
20	1y11mo	F	12	OTC	O(+)/ O(+)	Left lateral segment	Enteric fistula	Mother	Alive
21	3ys	F	13	CN-Ⅰ	B(+)/ B(+)	Left lateral segment	None	Mother	Alive

**Endpoints** Endpoints included: (1) patient and graft survival after transplantation; (2) major postoperative complications, such as lymphorrhea, bile leak, pulmonary infection, and acute or chronic rejection; (3) disease-specific parameters, including serum ceruloplasmin levels in Wilson disease, manifestations of portal hypertension in congenital hepatic fibrosis/Caroli disease, and metabolic crises in hereditary tyrosinemia type I; and (4) growth, assessed by changes in height, weight, and body mass index (BMI) Z-scores from baseline to 6 months after LDLT.

### Statistical analysis

Statistical analyses were performed using SPSS version 27.0. Continuous variables are presented as mean±standard deviation or median (interquartile range) and were compared using the independent-samples t-test or Mann–Whitney U test, as appropriate; categorical variables were compared using the chi-square test or Fisher’s exact test. Patient and graft survival were estimated by the Kaplan–Meier method and compared with the log-rank test. A two-sided P value < 0.05 was considered statistically significant. Height, weight and BMI Z-scores were calculated using the WHO anthropometric software(WHO Anthro/AnthroPlus); Z-scores < −2 and < −3 were defined as growth retardation and severe growth retardation, respectively, and Z-scores ≥ 0 indicated catch-up growth [5]. Within-person changes in growth Z-scores (pre-transplant vs. 6 months after LDLT) were evaluated using paired-samples t-tests (or Wilcoxon signed-rank tests as appropriate). Because WHO weight-for-age reference data are only available up to 10 years of age, weight-for-age Z-scores were calculated only in children < 10 years, whereas weight and BMI Z-scores were used in those aged ≥ 10 years.

## Results

### Patient characteristics

**Fig. 1:**
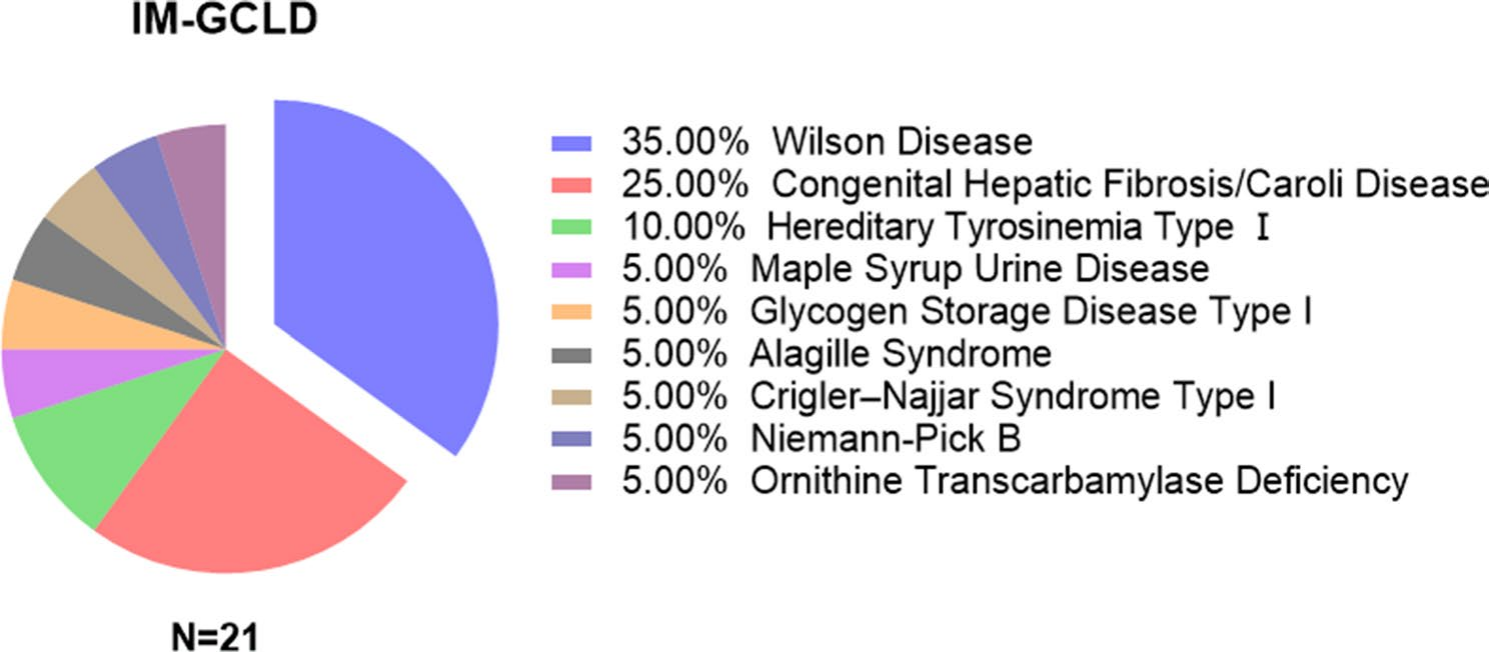
Distribution of patients according to their diagnoses

A total of 45 children underwent living donor liver transplantation, including 21 in the IM-GCLD group and 24 in the non-IM-GCLD group. The distribution of IM-GCLD subtypes is shown in Fig. 1, and baseline clinical characteristics are summarized in Table 2. The median age at transplantation was significantly higher in the IM-GCLD group than in the non-IM-GCLD group [108.0 (54.0–132.0) months vs. 7.0 (5.69–77.0) months, *P* < 0.001], reflecting the generally more indolent onset and progression of metabolic disorders compared with cholestatic diseases. The sex distribution did not differ significantly between the two groups. Pre-transplant weight and height Z-scores were similar, with no significant differences between groups. Neurologic and renal involvement were more frequent in the IM-GCLD group, mainly among children with Wilson disease and congenital hepatic fibrosis/Caroli disease. Pre-transplant PELD/MELD scores were comparable between the two groups, indicating a similar degree of hepatic dysfunction at the time of transplantation.

**Table 2:** Comparison of clinical characteristics between IM-GCLD and non-IM-GCLD pediatric patients

Characteristic	IM-GCLD group (*n* = 21)	Non IM-GCLD group (*n* = 24)	*P* value
Age(months)	108.00(54.00,132.00)	7.03(5.98,80.50)	< 0.001
Sex (male/female)	9/12	13/11	0.626
Preoperative weight Z-score	-0.65 ± 1.53	-1.32 ± 1.49	0.146
Preoperative height Z-score	-1.18 ± 2.17	-1.41 ± 2.50	0.752
Neurological and renal involvement	12/9	2/22	0.013
PELD/MELD	10.48 ± 9.16	14.83 ± 8.75	0.110

### Perioperative outcomes

**Fig. 2:**
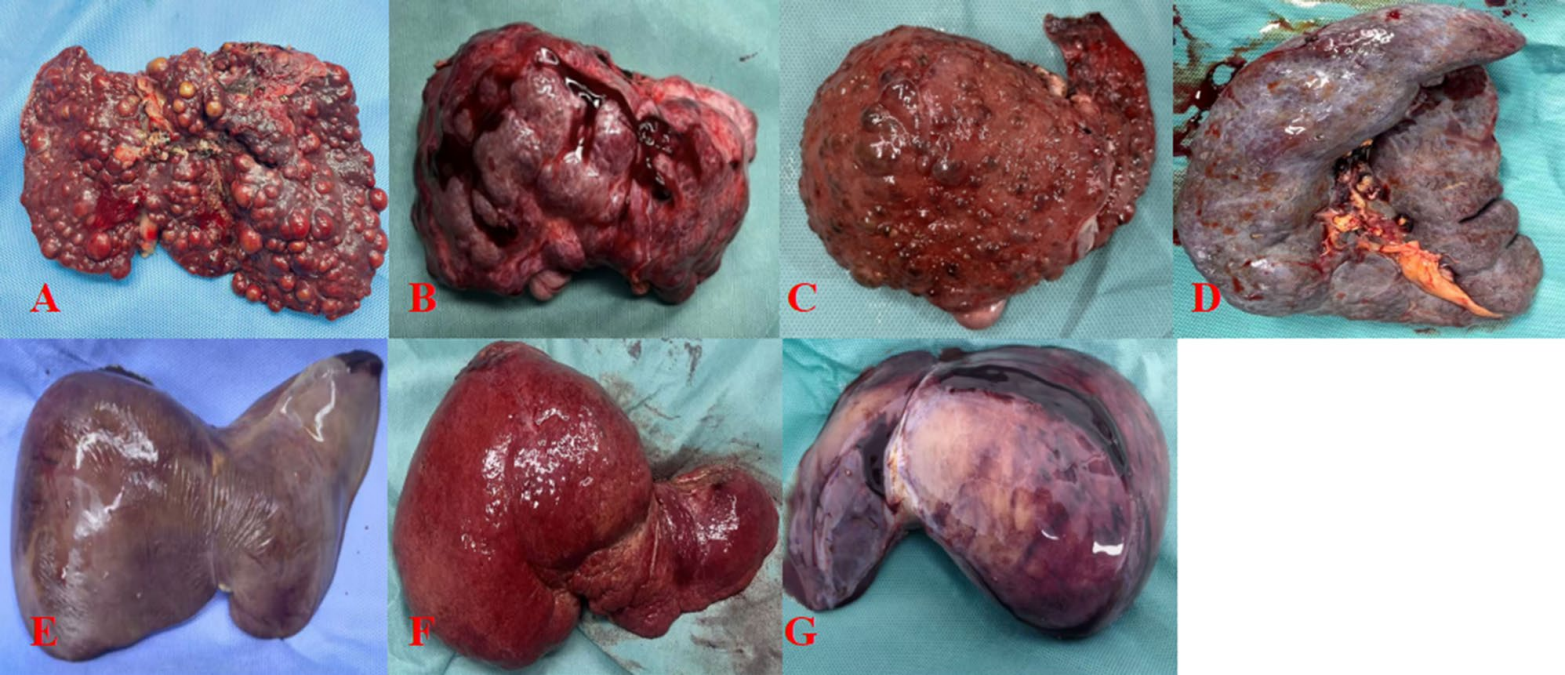
Representative images of explanted livers from pediatric patients with IM-GCLD; **A**: Wilson’s disease; **B**:Congenital hepatic fibrosis; **C**:Hereditary tyrosinemia type I ; **D**:Glycogen storage disease type I; **E**:Crigler–Najjar syndrome type I; **F**:Niemann–Pick disease type B; **G**:Ornithine transcarbamylase deficiency

In the IM-GCLD group (*n* = 21), the median operative time was 10.45 (9.05–12.71) hours, with a median warm ischemia time of 1.50 (1.00–2.00) minutes and a mean cold ischemia time of 79.79 ± 8.85 min. Median intraoperative blood loss was 750.00 (400.00–950.00) mL, and the graft-to-recipient weight ratio (GRWR) was 1.43 (0.89–1.91)(see Supplementary Table S2). Representative images of the explanted diseased livers are shown in Fig. 2.

**Fig. 3:**
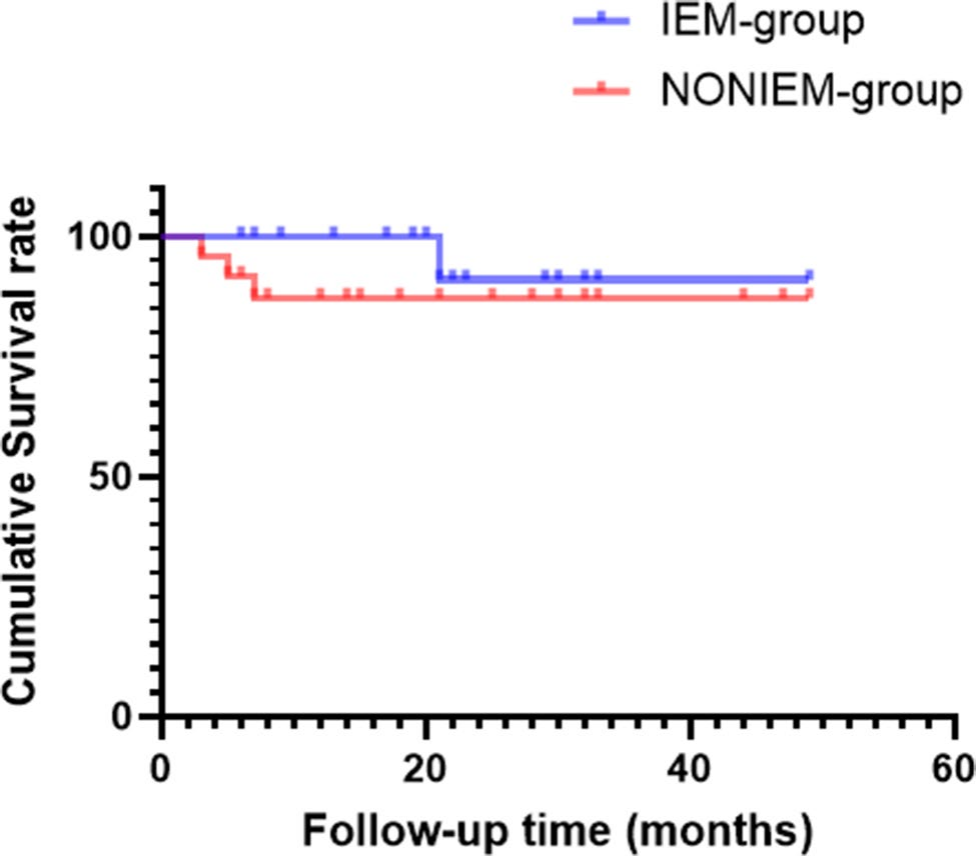
Post-transplant cumulative survival in IM-GCLD and non-IM-GCLD children

Postoperative complications occurred in eight children: one patient died in the perioperative period due to disseminated intravascular coagulation, and another died 21 months after transplantation from pneumonia-related multiorgan failure; lymphorrhea was observed in one patient, small-for-size syndrome in one, pulmonary infection in two, and chronic rejection in two. The median follow-up duration was 21 months (range, 6–49 months). Growth parameters at 6 months after LDLT were markedly improved in children with IM-GCLDs. There was no significant difference in cumulative survival between the IM-GCLD and non-IM-GCLD groups (Tables 3 and 4; Fig. 3).

**Table 3:** Weight and height z-scores before transplantation and at 6 months after LDLT in IM-GCLD children aged < 10 years

Age<10ys	Weight Z-score	Height Z-score
Pre-transplantation	-0.48 ± 1.69	-0.76 ± 2.06
Post-transplantation	0.43 ± 0.49	-0.01 ± 1.41
t-value	-3.012	-2.701
P-value	0.017	0.027

**Table 4:** Height and BMI Z-scores before transplantation and at 6 Months After LDLT in IM-GCLD Children Aged ≥ 10 Years

Age≥10ys	BMI Z-score	Height Z-score
Pre-transplantation	0.18 ± 1.10	-1.49 ± 2.07
Post-transplantation	0.52 ± 0.53	-0.53 ± 1.03
t-value	-1.198	-2.333
P-value	0.261	0.045

### Disease-specific baseline features, transplant indications and outcomes

Across all IM-GCLD subtypes, LDLT was associated with consistent disease-specific benefits, including correction of copper and nitrogen metabolism, relief of portal hypertension and cholestasis, and normalization or catch-up of growth and neurodevelopment (Table 5).

- WD (*n* = 8; age range 8–14 years): common features included hepatic failure with variable neuropsychiatric manifestations; transplantation was indicated for decompensated liver disease/fulminant hepatic failure or poor control [6, 7]. Post-LDLT, copper metabolism corrected and neurologic symptoms improved in most survivors; one perioperative DIC-related death occurred.
- CHF/CD (*n* = 5; age range 6–12 years): portal hypertension with GI bleeding/splenomegaly and/or biliary complications; transplantation was indicated for refractory portal hypertension complications and recurrent biliary complications; portal hypertension improved after LDLT [8, 9]. One CD recipient with polycystic kidney disease received a kidney-sparing immunosuppressive regimen (sirolimus plus mycophenolate mofetil) with close renal function monitoring.
- HT1 (*n* = 2; age range 1–2 years): metabolic crisis and liver failure; transplantation indicated for acute liver failure or high-risk course [10]; metabolic stability achieved after LDLT (one late death due to pneumonia-related MOF).
- Other rare IM‑GCLDs (each *n* = 1): MSUD (metabolic crisis), GSD I (hypoglycemia/hepatomegaly), ALGS (pruritus/cholestasis), CN-I (severe unconjugated hyperbilirubinemia), NPD-B(hepatomegaly/ascites), OTC (recurrent hyperammonemia). Disease-specific parameters normalized or improved after LDLT across these subtypes [11–15]. In addition, one biliary atresia recipient in the non-IM-GCLD cohort received a domino graft from the explanted MSUD liver; early graft function was satisfactory, but the postoperative course was complicated by severe infection and death.

A detailed individual-level summary of IM-GCLD recipients, including diagnosis, perioperative features, and post-transplant outcomes, is presented in Supplementary Table S1.

**Table 5:** Disease-specific baseline features, transplant indications and outcomes in IM-GCLD recipients

Disease subtype	*n*	Key pre-transplant features	Post-transplant outcomes	Growth Neurodevelopment	Remarks
WD	8	Hepatic failure, tremor	Ceruloplasmin normalized; neurologic symptoms improved in 7	Catch-up growth	One perioperative DIC death; chronic rejection(*n* = 2).
CHF/CD	5	GI bleeding, splenomegaly	Portal hypertension resolved	Growth improved	lymphatic leakage(*n* = 1) ;small-for-size syndrome(*n* = 1)
HT1	2	Metabolic crisis, liver failure	Metabolic stability; no recurrence	Normal growth	Postoperative pulmonary infection(*n* = 1)
MSUD	1	Feeding intolerance, metabolic crisis	Branched-chain amino acid levels normalized	Normal neurodevelopment	-
GSD I	1	Hypoglycemia, hepatomegaly	Glucose homeostasis restored	Catch-up growth	-
ALGS	1	Pruritus, cholestasis	Bile flow restored; pruritus relieved	Growth improved	Postoperative pulmonary infection(*n* = 1)
CN-I	1	Severe unconjugated hyperbilirubinemia	Bilirubin normalized	Normal development	-
NPD-B	1	Hepatomegaly, ascites	Liver function normalized	Stable growth	-
OTC	1	Recurrent hyperammonemia	Ammonia normalized; neurologic function improved	Normal development	-

## Discussion

In this single-center cohort, LDLT for heterogeneous IM-GCLDs was associated with favorable survival, with most children alive and maintaining stable graft function during follow-up. Importantly, the benefit of transplantation was not limited to survival. Across diagnostic categories, we observed clinically meaningful and largely consistent disease-specific improvements, including metabolic stabilization (e.g., correction of copper and nitrogen metabolism), relief of portal hypertension and cholestasis, and improved growth trajectories. These findings support LDLT as an important therapeutic option for selected IM-GCLDs and underscore the need for individualized perioperative assessment and long-term follow-up given phenotypic heterogeneity.

Our results are broadly consistent with published pediatric studies. Sanada and colleagues (2021) reported favorable outcomes after pediatric LDLT for inherited metabolic diseases and emphasized that transplantation can provide survival and developmental benefits across diverse diagnoses [16]. Registry-based evidence (e.g., OPTN/SRTR annual reports) further provides context for contemporary pediatric transplant outcomes and complication patterns [17]. Building on this literature, our study extends prior work by integrating both metabolic and genetic cholestatic conditions within a single cohort and by benchmarking IM-GCLD recipients against contemporaneous non-IM-GCLD recipients treated at the same center during the same period. Although baseline characteristics differed between groups (e.g., age distribution and extrahepatic involvement), overall survival was not significantly different, supporting the feasibility of LDLT in IM-GCLDs under individualized perioperative management. For rare diseases, survival alone may not capture the most meaningful clinical benefit; therefore, this integrated evaluation framework offers a more practical and clinically grounded interpretation of the real-world value of LDLT in IM-GCLDs.

Given one perioperative death related to disseminated intravascular coagulation (DIC), systematic perioperative coagulation assessment and dynamic monitoring are particularly important in selected IM-GCLD recipients presenting with severe liver failure. In the fatal case, preoperative testing demonstrated profound coagulopathy (PT 42.1 s, INR 4.30, APTT 80.7 s, fibrinogen 1.34 g/L, and platelet count 61 × 10⁹/L), and intraoperative fibrinolysis was markedly enhanced (D-dimer 4841 ng/mL; FDP 53.94 µg/mL), with substantial transfusion requirements (15 units RBC and 960 mL plasma, plus 501 mL autologous blood reinfused), indicating severe hemostatic derangement. Regarding monitoring, we propose a standardized pathway of “preoperative assessment+intraoperative reassessment+early postoperative re-evaluation”: preoperatively, at minimum PT/INR, APTT, fibrinogen, and platelet count should be obtained (with D-dimer/FDP when indicated); intraoperatively, these indices should be reassessed at key time points (hepatectomy phase, anhepatic phase, and post-reperfusion), while transfusion volumes are recorded in parallel; and in the early postoperative period, repeat testing should be performed to identify ongoing fibrinolysis or consumptive coagulopathy. Conventional coagulation assays provide limited real-time insight during transplantation; in contrast, viscoelastic testing (TEG/ROTEM) offers point-of-care, whole-blood assessment of clot formation and fibrinolysis and is better suited for phase-specific, goal-directed correction strategies [18, 19]. Although viscoelastic-guided algorithms may reduce transfusion in some settings, evidence is not uniform; therefore, phase-specific protocols should be implemented and overtreatment avoided.

In this cohort, growth recovery after LDLT was evident, particularly among younger children. Catch-up growth likely reflects improved hepatic function, enhanced nutrient absorption, and relief of cholestasis-related malnutrition; meanwhile, reduced metabolic disturbances and fewer episodes of metabolic stress after transplantation contribute to a more stable metabolic state, thereby promoting growth and development [20]. Our age-stratified descriptions (< 10 vs. ≥ 10 years) showed improvement in both strata, supporting the clinical relevance of transplantation timing and structured post-transplant nutritional rehabilitation. In older children, BMI-for-age Z-scores provide an age-appropriate assessment because WHO weight-for-age references are limited to children < 10 years.

In our cohort, parental consanguinity was documented in 10 cases, accounting for 55.6% of the IM-GCLD patients. This proportion is markedly higher than that in the general population and suggests that consanguineous marriage may be an important contributing factor to the high burden of inherited metabolic liver diseases (IEMs) in Xinjiang. Previous studies have demonstrated that consanguinity significantly increases the risk of autosomal recessive disorders, leading to a higher prevalence of IEMs in populations with elevated rates of consanguineous unions [21]. These findings underscore the importance of strengthening premarital and prenatal genetic counseling and public health education to help reduce the incidence of such disorders.

Limitations of this study include its retrospective single-center design, the limited sample size for each diagnostic subtype, and short-to-moderate follow-up, which restrict definitive inference for individual rare subtypes and limit the feasibility of multivariable analyses. Larger multicenter cohorts and registry-based studies are needed to validate diagnosis-specific trajectories and long-term outcomes.

## Conclusions

Our findings demonstrate that LDLT not only ensures favorable overall survival in children with IM-GCLDs but also confers substantial disease-specific benefits across different subtypes, including recovery of neurologic function, restoration of metabolic homeostasis, relief of portal hypertension, improvement of cholestasis, and catch-up growth. For autosomal recessive IM-GCLDs, the use of heterozygous parental donors appeared to be safe and clinically effective in most cases. Taken together, these results indicate that LDLT is not merely a life-saving intervention for children with IM-GCLDs, but also a key strategy for achieving long-term remission and meaningful improvement in quality of life.

## Supplementary Information

Below is the link to the electronic supplementary material.

Supplementary Material 1

Supplementary Material 2

## Data Availability

The data presented in this study are not publicly available due to ethical and privacy restrictions involving pediatric patients. De-identified datasets are available from the corresponding author on reasonable request and with approval from the institutional ethics committee.

## Abbreviations

IM-GCLDs: Inherited metabolic and genetic cholestatic liver diseases
LDLT: Living donor liver transplantation
WD: Wilson disease
CHF: Congenital hepatic fibrosis
CD: Caroli disease
HT1: Hereditary tyrosinemia type I
MSUD: Maple syrup urine disease
GSD I: Glycogen storage disease type I
ALGS: Alagille syndrome
CN-I: Crigler–Najjar syndrome type I
NPD-B: Niemann–Pick disease type B
OTC: Ornithine transcarbamylase deficiency
PELD: Pediatric End-Stage Liver Disease score
MELD: Model for End-Stage Liver Disease score
IEMs: Inherited metabolic liver diseases
