# Supplementary material for: Living-donor liver transplantation in children with inherited metabolic and genetic cholestatic liver diseases: a single-center retrospective cohort study
Source: Orphanet J Rare Dis. 2026 Apr 30;21:183. doi: 10.1186/s13023-026-04369-4 (PMC13154865; doi:10.1186/s13023-026-04369-4)
Supplement: Supplementary file 1 — Supplementary Material 1 [file 13023_2026_4369_MOESM1_ESM.docx]

Table S1 Clinical features and outcomes of IM-GCLD recipients after living donor liver transplantation.

| NO. | Age | Sex | Weight (kg) | Diagnosis | 6-Month Growth Trend | Major Post-Transplant Complications | Status |
| --- | --- | --- | --- | --- | --- | --- | --- |
| 1 | 14ys | M | 40 | WD | Catch-up | None | Alive |
| 2 | 11ys | F | 37 | WD | Improved | None | Alive |
| 3 | 1y | M | 6.5 | HT-Ⅰ | Catch-up | Pulmonary infection | Deceased |
| 4 | 10ys | F | 33 | CHF | Stable | Lymphatic leakage | Alive |
| 5 | 8ys | M | 29 | CHF | Stable | None | Alive |
| 6 | 8ys | M | 38 | WD | Stable | Chronic rejection | Alive |
| 7 | 9ys | F | 30 | WD | Stable | Disseminated intravascular coagulation | Deceased |
| 8 | 6ys | F | 23 | CHF | Stable | None | Alive |
| 9 | 9mo | M | 6 | MSUD | Catch-up | None | Alive |
| 10 | 12ys | F | 38 | CHF | Stable | Small-for-size syndrome | Alive |
| 11 | 10ys | M | 27 | CD | Catch-up | None | Alive |
| 12 | 11ys | M | 34 | WD | Stable | None | Alive |
| 13 | 9ys | F | 30 | WD |  | None | Alive |
| 14 | 10ys | F | 37 | WD | Stable | Chronic rejection | Alive |
| 15 | 10ys | F | 24 | GSD-Ⅰ | Catch-up | None | Alive |
| 16 | 2ys | M | 10 | HT-Ⅰ | Catch-up | None | Alive |
| 17 | 9ys | M | 23 | ALGS | Improved | Pulmonary infection | Alive |
| 18 | 12ys | F | 39 | WD | Stable | None | Alive |
| 19 | 13ys | F | 35 | NPD-B | Catch-up | None | Alive |
| 20 | 1y11mo | F | 12 | OTC | Stable | Enteric fistula | Alive |
| 21 | 3ys | F | 13 | CN-Ⅰ | Stable | None | Alive |
