# Supplementary material for: Living-donor liver transplantation in children with inherited metabolic and genetic cholestatic liver diseases: a single-center retrospective cohort study
Source: Orphanet J Rare Dis. 2026 Apr 30;21:183. doi: 10.1186/s13023-026-04369-4 (PMC13154865; doi:10.1186/s13023-026-04369-4)
Supplement: Supplementary file 2 — Supplementary Material 2 [file 13023_2026_4369_MOESM2_ESM.docx]

Table S2 Intraoperative Data of the 21 Pediatric Patients.

| Intraoperative Records | GRWR(%) | Operative time (h) | Blood loss (mL) | Cold ischemia time(min) | Warm ischemia time(min) |
| --- | --- | --- | --- | --- | --- |
| Total | 1.43(0.89,1.91) | 10.45(9.05,12.71) | 750.00(400.00,950.00) | 79.79±28.85 | 1.50(1.00,2.00) |
